# All-terrain vehicle (ATV)-related injuries among different age groups: insights from a 9-year observational study

**DOI:** 10.1007/s00068-022-01984-1

**Published:** 2022-05-21

**Authors:** Husham Abdelrahman, Naushad Ahmad Khan, Ayman El-Menyar, Rafael Consunji, Mohammad Asim, Mushrek Alani, Adam Shunni, Abubaker Al-Aieb, Hassan Al-Thani

**Affiliations:** 1 Trauma Surgery Section, Hamad Medical Corporation & Weill Cornell Medical College, Hamad General Hospital (HGH), PO Box 3050, Doha, Qatar; 2 Clinical Research, Trauma and Vascular Surgery Section, HGH, Doha, Qatar; 3 Clinical Medicine, Weill Cornell Medical College, Doha, Qatar; 4 Trauma Surgery Section, Hamad Injury Prevention Program, HGH, Doha, Qatar

**Keywords:** All-terrain vehicle (ATV), Trauma, Injury, Pediatrics, Age

## Abstract

**Purpose:**

To describe the epidemiology, patterns, seasonality and outcome of all-terrain vehicle-related injuries (ATVRIs) among different age groups in Qatar.

**Methods:**

A retrospective analysis of all patients hospitalized with ATVRI from 2010 to 2018 was conducted. Data were analyzed by age, gender, seasonality, and Injury severity Score  (ISS). Chi-square, Student t and ANOVA tests were used for analysis. Multivariate regression analysis was performed to find out predictors of head injury and severe injury among ATV users.

**Results:**

Out of 15,000 trauma admissions, 521 had ATVRI (4%) with a mean age of 23.3 ± 12.3. The male-to-female ratio was 4:1 and the pediatric population represented 40%. The compliance with helmet use was 3.6%. The most injured regions were chest (29.8%), upper extremities (28.8%) and the head (25.9%). The mean ISS was 10.6 ± 7.7. Fracture fixation was the most operative intervention. Among hospitalized patients, 7.7% were transferred to rehabilitation . ATVRIs occurred more frequently between October and April and a large proportion (57.8%) occurred during weekends. The overall ATV-related hospital mortality rate was 2.1%. Young and older ATV users were more likely to suffer spine injuries than the pediatric population (*p* = 0.001). The mean ISS was greater in the older groups (*p* = 0.03). There were no statistically significant differences regarding the use of protective devices, mortality, or length of hospital stay between the different age groups. On multivariate analysis, young age and ISS were predictors of head injury among ATV users after adjusting for gender and helmet use.

**Conclusion:**

This is a nationwide study looking at all age groups who sustained ATVRI in Qatar. ATVRIs were observed in all age groups following leisure and recreational use. It follows a seasonal pattern with poor protective measures compliance. There is a need to reinforce helmet use and raise public awareness.

**Supplementary Information:**

The online version contains supplementary material available at 10.1007/s00068-022-01984-1.

## Introduction

Quad bikes or all-terrain vehicles (ATVs) have been used by the public for farming, ranching, hunting, and other agricultural use, as well as for recreation and sports use, for over 40 years [1, 2]. The popularity of ATV is because they are fast, powerful, off-road vehicles with excellent maneuverability and applicability to many tasks [1]. The use of ATV has also increased the recreational utility of a vehicle with a high center of gravity and a challenging need for constant adjustments while operating it. This has led to an increase in ATV-related injuries, which can be attributed to a lack of proper training related to vehicle operation and safety compliance [3].

Risk factors for ATV injury have been reported from western and high-income countries. One of these is engine displacement, with a growing demand for more powerful and faster models [1, 2]. Other factors include the lack of public ATV safety awareness, specific preventive measures, and legislation for the mandatory use of helmets, harnesses, and matching the engine displacement to the user size and capacity to control the vehicle [1, 4, 5].

Unfortunately, the pediatric population represents a high-risk group for ATV-related injuries (ATVRI). Much has been reported on severe and fatal ATVRI and the resultant life-long disability [6–9]. Children below 16 years of age are particularly at risk for ATVRIs, with risky behavior and the use of high-powered ATVs designed for adults [9, 10]. Moreover, the lack of physical power, incomplete neuromotor development, and limited cognitive capacity for younger children increase their risk for ATVRI as precocious ATV users [11]. Despite a growing body of literature related to ATVRIs in youth [12–14], none have specifically compared the risk of moderate-to-severe injuries across different age groups, especially in the younger population in the Middle Eastern region. Most of the reports of ATV-related injuries came from North America, Australia, and the UK [15].

In recent years, the popularity of ATVs has skyrocketed in Qatar as a recreational sport, especially on the weekends [16]. Over the last decade, there has been an increase in ATV-associated awareness among the public, media, and health care professionals [3, 17–19]. However, the only report on Qatar’s ATV injuries was based on a limited population of 56 patients presented during the first 10 months of a new trauma service in 2008. The use of ATV is mainly for leisure in Qatar and represents an important mode of head trauma [16, 20]. Descriptions of risk and contributing factors and burdens from ATVRIs are needed to provide evidence for the formulation of ATV safety laws, public awareness campaigns, and the implementation of preventive programs. The primary objective of the current study is to describe the incidence, demographics, injury patterns, outcomes among different age groups, and assess the impact of ATV use over 9 years in Qatar. Also, we explored the role of age, use of helmets, and the seasonal/weekend effect on the frequency of ATVRIs. Overall, we aim to provide data to inform and support the ongoing public awareness campaigns and prevention programs to decrease ATV-related morbidity and mortality in the country.

## Methods

### Setting and data source

A retrospective cohort analysis was conducted on a prospectively maintained database that included all consecutive victims of mild to severe ATVRIs, from the national trauma registry of Hamad Trauma Center [HTC]. The HTC is the only Level I trauma center serving the entire country, receiving more than 98% of the county’s trauma patients. The trauma registry at the study institution has regular internal and external validations and is compliant with both the National Trauma Data Bank [NTDB] and Trauma Quality Improvement Program [TQIP] of the American College of Surgeons-Committee on Trauma. The HTC consists of four major units for patient care, i.e. trauma resuscitation unit (TRU), trauma intensive care unit (TICU), trauma in-patient ward (TSU), and trauma outpatient clinic. The TRU consists of five trauma bays and is equipped with state-of-the-art technology including ground as well as air ambulance service which can bring injured patients directly to the TRU for in-hospital care, the TICU is a 19-bed critical care unit and the TSU has 20-bed capacity in-patient ward.

Injuries were defined according to the International Classification of Diseases, Ninth Revision (ICD-9) codes between 800 and 959.9. All trauma patients with trauma activation who are admitted to the trauma service have an ICD-9 code: E821.0. The All-Terrain Vehicle Industry European Association defines an ATV as “any motorized vehicle designed to travel on four low-pressure tires on unpaved surfaces, having a seat designed to be straddled by the operator and handlebars for steering control” [21].

### Patients

The study population included all trauma patients requiring hospitalization to the trauma surgery service with injuries resulting from ATV crashes between 2010 and 2018. Exclusion criteria included patients who were dead on arrival or patients with incomplete relevant data.

### Variables and outcomes

Collected data included patient demographics (age, gender, nationality), helmet use, alcohol consumption, type of injury [head, chest, abdomen, pelvis, spine, and extremities (upper and lower)] with corresponding abbreviated injury score (AIS), Injury severity score (ISS), Severity categories according to ISS (mild < 9, moderate 9–15 and severe > 15), revised trauma score (RTS), patient disposition and outcome. Patients were stratified by age into pediatric (≤ 18 years) and adults (> 18 years old). The groups were < 10, 10–18, 19–30, 31–40, 41–50 and > 50 years old. Furthermore, we looked at the time of the injury. The outcomes of interest were overall in-hospital mortality, complications (sepsis, Ventilator-Associated Pneumonia; VAP), and hospital course (i.e. length of stay).

Ethical approval for this study was obtained from Research Ethics Committee at Medical Research Center (MRC-01-18-473), Hamad Medical Corporation (HMC), Doha, Qatar.

## Statistical analysis

Data were interpreted as a proportion or mean ± standard deviation or mean and 95% confidence interval or median and interquartile range e whenever applicable. Comparisons between 2 and more groups were made using chi-square for categorical variables. For continuous variables comparing two groups, Student *t* test was used, and one-way ANOVA was performed for more than two groups. A bivariate analysis was performed to compare the injury characteristics, injury severity and outcomes of ATV-related causalities stratified by pediatric and adult subjects (age ≤ 18 vs age > 18). The unadjusted odds ratio (OR) and 95% confidence interval (CI) for univariate analysis was also reported. For predictors of head injury among ATV users, multivariate logistic regression analysis was performed using relevant variables such as age, gender, ISS and use of helmet. Data were expressed using OR and 95% CI. A two-tailed *p* value of < 0.05 signifies statistical significance. Data analysis was carried out using the Statistical Package for Social Sciences version 21 (SPSS Inc., Chicago, IL) and Graphpad Prism (GraphPad Software, San Diego, CA). This Paper follows the STrengthening the Reporting of OBservational studies in Epidemiology (STROBE) (Suppl file).

## Results

### Overall

A total of 521 (4.3% of all admissions) ATVRIs were identified. Overall, the study population was predominantly young, with a mean age of 23.3 ± 12.3 years. Adults accounted for 59.8% of the admissions (*n* = 312), and children (age ≤ 18), accounted for 39.3% (*n* = 205). Most patients were males (80.2%), and the male to female ratio was 4:1. Only 3.6% of patients (*n* = 19) used helmets while riding ATVs. Table 1 describes the demographics, pattern of associated injuries and complications, comorbidities, injury patterns, severities, interventions, and outcome measures of ATV-related injuries. The most injured regions were the chest (29.8%) and upper extremity (28.8%), followed by the head (25.9%), spine (21.7%), lower extremity (15%), abdomen (15%), and pelvis (9.8%) respectively. Also, one-fifth of the ATVRI cases had polytrauma (ISS > 15).

**Table 1:** Demographics, clinical characteristics, associated injuries, complications, injury characteristics, severities and outcomes of ATV related causalities in the study (*n* = 521)

Variables	Value	Variables	Value
Age, (mean ± SD)	23.3 ± 12.3	Pelvis AIS, (mean ± SD)	2.2 ± 0.5
Gender		Leg AIS, (mean ± SD)	2.5 ± 0.5
Male (*N*%)	418 (80.2%)	ISS (mean ± SD)	10.6 ± 7.7
Protective devices		ISS > 15 (*N*%)	109 (21.2%)
Yes (*N*%)	19 (3.6%)	RTS, (mean ± SD)	7.6 ± 0.8
No (*N*%)	502 (96.4%)	Initial vitals at ED	
Associated injuries		Pulse, (mean ± SD)	93.7 ± 19.7
Head injury (*N*%)	135 (25.9%)	SBP, (mean ± SD)	125.9 ± 18.9
Chest injury (*N*%)	155 (29.8%)	DBP, (mean ± SD)	74.8 ± 12.8
Abdominal injury (*N*%)	77 (14.8%)	GCS (median and IQR)	15 (3–15)
Upper extremity (*N*%)	150 (28.8%)	Interventions	
Spine injury (*N*%)	113 (21.7%)	Exploratory laparotomy (*N*%)	9 (1.7%)
Pelvis injury (*N*%)	51 (9.8%)	Thoracotomy (*N*%)	3 (0.6%)
Lower extremity (*N*%)	78 (15%)	External fixation (*N*%)	15 (2.9%)
Comorbidities		Closed reduction (*N*%)	30 (5.8%)
Diabetes (*N*%)	14 (2.7%)	ORIF (*N*%)	85 (16.3%)
Hypertension (*N*%)	10 (1.9%)	Craniectomy (*N*%)	10 (1.9%)
Heart disease (*N*%)	8 (1.5%)	ICP (*N*%)	6 (1.2%)
Complications		Operation of spine (*N*%)	11 (2.1%)
Sepsis (*N*%)	2 (0.4%)	Chest tube (*N*%)	37 (7.1%)
ARDS (*N*%)	1 (0.2%)	MTP (*N*%)	10 (1.9%)
VAP (*N*%)	7 (1.3%)	Intubation	55 (10.6%)
SSI (*N*%)	1 (0.2%)	Discharge disposition	
Ethanol positive^a^ (*N*%)	10 (2.2%)	Discharged to home (*N*%)	469 (90.2%)
Abbreviated injury scale (AIS)		Discharged to rehabilitation (*N*%)	13(2.5%)
Head AIS, (mean ± SD)	3.2 ± 0.9	Transferred to other facility (*N*%)	27 (5.2%)
Head AIS ≥ 3^b^ (*N*%)	112 (86.2%)	Ventilatory days	3 (1–25)
Chest AIS, (mean ± SD)	2.7 ± 0.7	ICU days, median (range)	3 (1–35)
Abdomen AIS (mean ± SD)	2.3 ± 0.8	HLOS, median (range)	4 (1–68)
Arm AIS, (mean ± SD)	2.1 ± 0.4	Outcome (mortality) (*N*%)	11 (2.1%)
Spine AIS, (mean ± SD)	2.2 ± 0.6		

*ARDS* acute respiratory distress syndrome, *VAP* Ventilator-Associated Pneumonia, *SSI* surgical site infections, *ISS* injury severity score, *RTS* Revised Trauma Score, *ED* emergency department, *SBP* systolic blood pressure, *DBP* diastolic blood pressure, *GCS* Glasgow Coma Score, *ORIF* open reduction and internal fixation, *ICP* intra cranial pressure, *MTP* massive transfusion protocol, *ICU* intensive care unit, *HLOS* hospital length of stay

^a^Data are available for 438 cases

^b^Data for head AIS are available for 130 cases

Positive blood alcohol levels were reported in 10 (2.3%) of 438 cases, who met the criteria for blood alcohol testing, and the ethanol level ranged from 4 to 78 with a median value of 37 (The HTC policy is mandatory alcohol testing for trauma patients who are 12 years old and above). The mean ISS was 10.6 ± 7.7. Severe injuries (ISS > 15) were reported in 21% of the subjects (*n* = 109), and 112 (86.3%) of patients with a head injury had a severe head injury, i.e., Head AIS ≥ 3. Fifty-five patients were intubated (11%), and the median ICU stay was three (1–35) days.

### Interventions

Chest tube insertion was done in 37 (7%), while ICP was performed in 1.2%, and three patients needed a thoracotomy. Nearly 40% underwent a surgical procedure; the most common was ORIF in 85 (16%), while 30 (6%) underwent closed reduction and external fixation in 15 (3%). The other operative interventions included spine fixation in 11 (2%), craniectomy in 10 (2%), and exploration laparotomy in 9 (2%) subjects.

### Outcomes

Overall mortality was 2% (11 patients). The disposition of patients included 90.2% discharged to home, 5.2% were transferred to another facility, and 2.5% moved to rehabilitation care.

Table 2 compares the characteristics of adults versus pediatric ATVRIs. Adult were less likely to have a head injury when compared to children [OR:0.57 (0.38–0.85); *p* < 0.001], and the reverse was true for spine injuries [OR:3.42 (2.08–5.58); *p* < 0.001]. There was a significantly higher proportion of adult female victims in comparison to males (22% vs 16% respectively) and fewer chest injuries in the pediatric population. In comparison to children, adults had a substantial difference in the median ventilator days and duration of ICU (p<0.001) ,but the overall hospital stay was comparable.

**Table 2:** Injury characteristics, severities and outcomes of ATV related causalities stratified by pediatric and adult subjects (*n* = 517)

Variables	Age ≤ 18 *N* = 221	Age > 18 *N* = 296	OR (95%CI)	*p* value
Gender				
Male (*N*%)	186 (84.2%)	229 (77.4%)	1.55 (0.98–2.44)	0.05
Female (*N*%)	35 (15.8%)	67 (22.6%)	1 (Ref)	
Associated injuries				
Head injury (*N*%)	70 (31.7%)	62 (20.9%)	0.57 (0.38–0.85)	0.001
Chest injury (*N*%)	69 (31.2%)	85 (28.7%)	0.88 (0.61–1.29)	0.53
Abdominal injury (*N*%)	37 (16.7%)	39 (13.2%)	0.75 (0.46–1.220	0.25
Upper extremity (*N*%)	54 (24.4%)	96 (32.4%)	1.48 (1.00–2.19)	0.04
Spine injury (*N*%)	24 (10.9%)	87 (29.4%)	3.42 (2.08–5.58)	0.001
Pelvis injury (*N*%)	21 (9.5%)	30 (10.1%)	1.07 (0.59–1.930	0.81
Lower extremity (*N*%)	37 (16.7%)	41 (13.9%)	0.80 (0.49–1.29)	0.36
ISS (Injury Severity Score), (mean ± SD)	10.02 ± 7.72	10.76 ± 7.17	–	0.67
Ventilatory days, median (range)	1 (1–25)	5 (1–23)	–	0.03
Intensive care unit (ICU) days, median (range)	2 (1–35)	5 (1–34)	**–**	0.001
Hospital length of stay (HLOS), median (range)	4 (1–50)	4 (1–68)	–	0.93
Mortality (*N*%)^a^	2 (0.9%)	6 (2.0%)	2.26 (0.45–11.33)	0.30

^a^Age missing in four cases

### Distribution of ATVRI among different age groups

**Fig. 1:**
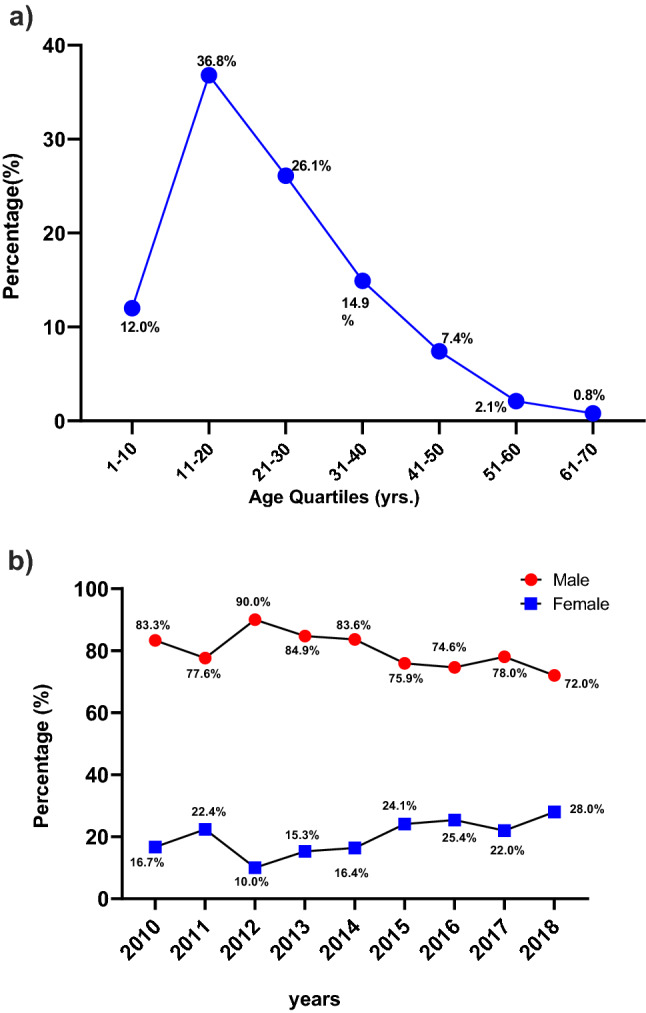
**a** ATV-related injuries by age group. **b** ATV-related injuries by age group

When stratified by age group, most injuries occurred in the 11–20 years (36.8%) and 21–30 years (26.1%) old groups, with the majority of victims being male (82% in 1–20 years and 73% in 21–30 years). Few ATVRIs affected those above 40 years of age (Fig. 1a). Figure 1b outlines the breakdown of pediatric patients (*n* = 205) stratified into 5-year age intervals; the 11–14-year-old was found to be the highest risk group for ATVRI, accounting for nearly half of the pediatric subjects (48.9%), this was followed by the 15–18 years-old group (30%). Table 3 shows the characteristics of ATV injured in different age groups. The pediatric population (< 10 and 10–18 years old) was more likely to present with a head injury compared to the adult population (*p* = 0.001). The older ATV users were more likely to have upper extremity injury than the young and pediatric population *p* = 0.02). Young (>18 - < 50 years) and older ATV (> 50 years) users were more likely to suffer spine injuries compared to the pediatric population (*p* = 0.001). The mean Injury severity scores increased significantly with increasing age groups (*p* = 0.03). There were no statistically significant differences regarding the use of protective devices, mortality, or length of hospital stay between the different age groups.

**Table 3:** Injury characteristics, severities and outcomes of ATV related causalities stratified by different age subgroup

Variables	Age group (years)						*p* value
	< 10 (*n* = 45)	10–18 (*n* = 176)	19–30 (*n* = 166)	31– 40 (*n* = 77)	41–50 (*n* = 38)	> 50 (*n* = 15)	
Males	41 (91.1%)	145 (82.4%)	124 (74.7%)	63 (81.8%)	29 (76.3%)	13 (86.7%)	0.15
Protective devices	2 (4.4%)	10 (5.7%)	4 (2.4%)	2 (2.6%)	1 (2.6%)	0 (0%)	0.58
Head injury	17 (37.8%)	53 (30.1%)	43 (25.9%)	5 (6.5%)	10 (26.3%)	4 (26.7%)	0.001
Chest injury	13 (28.9%)	56 (31.8%)	50 (30.1%)	24 (31.2%)	11 (28.9%)	0 (0%)	0.23
Abdominal injury	7 (15.6%)	30 (17%)	23 (13.9%)	12 (15.6%)	2 (5.3%)	2 (13.3%)	0.59
Upper extremity	4 (8.9%)	50 (28.4%)	48 (28.9%)	28 (6.4%)	15 (39.5%)	5 (33.3%)	0.02
Spine injury	0 (%)	24 (13.6%)	48 (28.9%)	24 (31.2%)	11 (28.9%)	4 (26.7%)	0.001
Pelvis injury	1 (2.2%)	20 (11.4%)	16 (9.6%)	8 (10.4%)	5 (13.2%)	1 (6.7%)	0.53
Lower extremity	11 (24.4%)	26 (14.8%)	26 (15.7%)	9 (11.7%)	5 (13.2%)	1 (6.7%)	0.44
Head AIS, (mean ± SD)	3 ± 0.6	3.3 ± 0.8	3.3 ± 0.9	3 ± 0,	3.2 ± 1.1	3 ± 1.6	0.88
Head AIS ≥ 3	12 (80%)	46 (88.5%)	37 (88.1%)	5 (100%)	7 (77.8%)	3 (75.0%)	0.75
ISS, (mean ± SD)	8.6 ± 5.8	10.3 ± 7.9	11.1 ± 7.4	8.9 ± 5.1	13 ± 9.2	12 ± 8.6	0.03
ISS > 15	6 (13.6%)	43 (24.6%)	38 (23.2%)	8 (10.4%)	6 (16.2%)	5 (33.3%)	0.05
RTS, (mean ± SD)	7.4 ± 1.1	7.6 ± 0.9	7.6 ± 0.9	7.8 ± 0.4	7.8 ± 0.2	7.6 ± 0.9	0.21
GCS, (mean ± SD)	13.8 ± 3.5	13.9 ± 3.0	13.8 ± 3.2	14.7 ± 1.9	14.0 ± 2.9	14.2 ± 3.1	0.54
Exploratory laparotomy	3 (6.7%)	2 (1.1%)	3 (1.8%)	0 (0%)	0 (0%)	0 (0%)	0.07
ORIF	3 (6.7%)	28 (15.9%)	26 (15.7%)	17 (22.1%)	9 (23.7%)	2 (13.3%)	0.25
MTP	0 (%)	1 (0.6%)	6 (3.6%)	1 (1.3%)	1 (2.6%)	0 (0%)	0.29
Intubation	5 (11.1%)	19 (10.8%)	21 (12.7%)	2 (2.6%)	3 (7.9%)	1 (6.7%)	0.25
Hospital LOS, median (range)	3 (1–16)	4 (1–50)	4 (1–68)	4 (1–67)	4 (1–64)	3 (1–25)	0.15
Mortality	1 (2.2%)	1 (0.6%)	4 (2.4%)	0 (0%)	2 (5.3%)	0 (0%)	0.22

Age was not documented for four patients

*AIS* Abbreviated Injury Scale, *ISS* Injury Severity Score, *RTS* Revised Trauma Score, *GCS* Glasgow Coma Score, *ORIF* Open Reduction and Internal Fixation, *MTP* Massive transfusion Protocol, *LOS* Length of Stay.

### Injury severity among ATVRIs

**Fig. 2:**
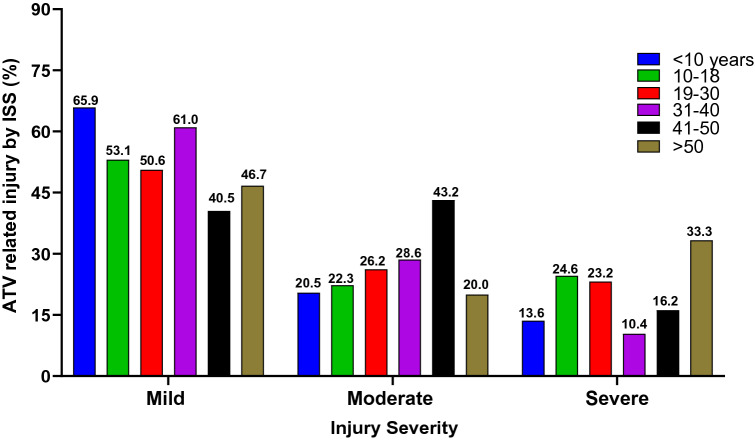
The proportion of ATV-related injuries stratified by injury severity defined by injury severity score (ISS)

Table 4 outlines the comparison between the different injury severities. The more severe the injury, the higher the incidence of head and abdominal injuries, the need for massive transfusion, and the rate of complications. Figure 2 depicts the proportion of ATV-related injuries stratified by injury severity defined by ISS. Data indicated that mild injury was more frequent in the pediatric population than in the adult population. Pediatric patients were less likely to be admitted with severe injury than the adult population. While much attention has been on pediatric trauma associated with ATV use, our data suggested that these vehicles are equally unsafe for riders of all ages.

**Table 4:** Injury characteristics, severity, and outcomes of ATVRIs, stratified by Injury Severity Score (ISS)

Variables	Injury Severity Score (ISS) *N* = 515^b^			*p*-value
	ISS < 9 (*N* = 274)	ISS ≥ 9–15 (*N* = 132)	ISS > 15 (*N* = 109)	
Age (mean ± SD)	22.67 ± 12.10	25.05 ± 12.64	22.98 ± 12.21	0.175
Male (*N*; %) (413; 80.2%)	215 (78.5%)	108 (81.8%)	90 (82.2%)	0.571
Female (*N*; %) (102; 19.8%)	59 (21.5%)	24 (18.2%)	19 (17.4%)	
Body mass index (BMI), (mean ± SD)	23.87 ± 5.86	25.91 ± 6.66	24.89 ± 5.65	0.144
Chest injury (*N*; %)	75 (27.4%)	43 (32.6%)	34 (31.2%)	0.510
Head injury (*N*; %)	21 (7.7%)	35 (26.5%)	75 (68.8%)	0.001
Abdominal injury, (*N*; %)	31 (11.3%)	20 (15.2%)	26 (23.9%)	0.008
Ethanol positive (*N*; %)	8 (2.9%)	1 (0.8%)	1 (0.9%)	0.229
Ventilator Associated Pneumonia (VAP) (N; %)^a^	0/4 (0.0%)	1/8 (12.5%)	6/43 (14.0%)	0.910
Revised Trauma Score, (RTS) (mean ± SD)	7.82 ± 0.19	7.70 ± 0.62	6.94 ± 1.50	0.001
Intensive Care unit (ICU) days, median (range)	2 (1–34)	2 (1–12)	4 (1–35)	0.02
Ventilatory days, median (range)	–	1 (1–3)	4 (1–25)	0.003
Hospital length of stay (HLOS) (median, range)	3 (1–67)	4 (1–41)	10 (1–68)	0.001
Mortality, (*N*; %)	0 (0.0%)	1 (0.8%)	6 (5.5%)	0.001

^a^The denominator represents the number of mechanically ventilated patients

^b^ISS missing in five cases

### Timings of ATVRIs

**Fig. 3:**
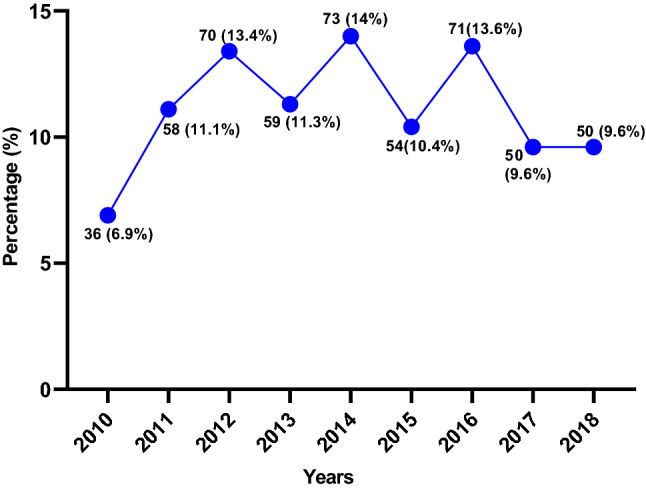
ATV-related injuries treated at Hamad Trauma Center, per year (2010–2018)

**Fig. 4:**
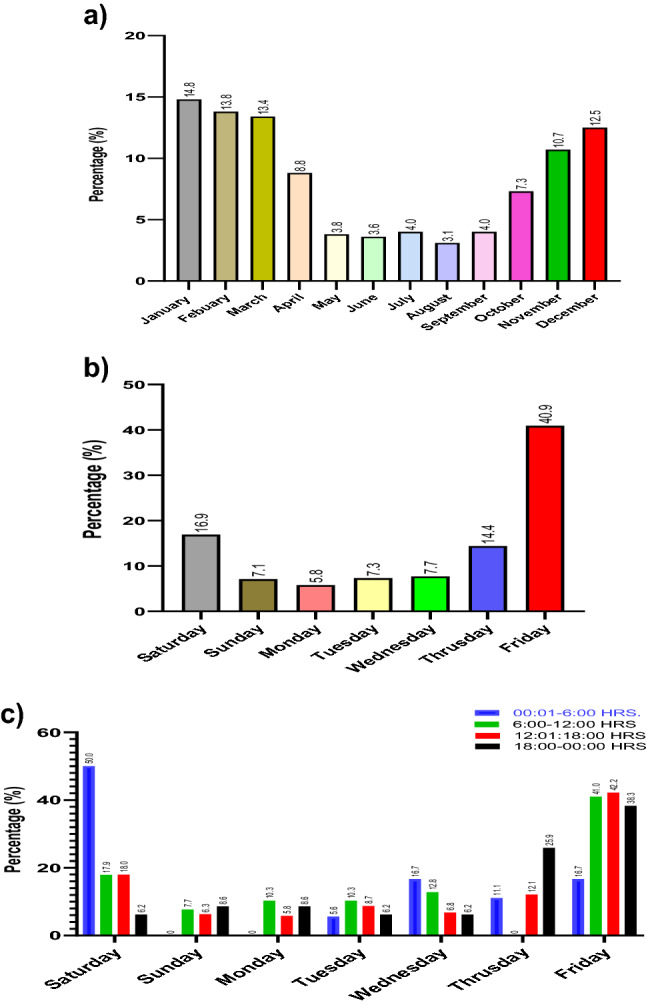
**a** Mean monthly incidence of ATV-related injuries; **b** mean daily incidence of ATV related injuries; **c** hourly incidence of ATV-related injuries

The annual average number of ATVRIs was 57.8. The peak of ATVRIs was observed in 2014 with 73 (14%) ATVRIs, but no apparent time trend was seen (Fig. 3). A decreasing trend was noted from 2017 through 2018 (*n* = 50 annually). There was a seasonal pattern of incidence of injury and more cases presented during weekends. The incidence of ATVRIs dropped during the hot summer months and showed a marked increase during the cooler months (October–March). Though ATVRI occurred throughout the year, the most injuries (80.3%) were seen from October to April, with a mean number of injuries per month being 11.6 (Fig. 4a). The overall mean number of injuries per day is demonstrated in Fig. 4b. The weekends in Qatar are Fridays and Saturdays. A large proportion of the ATVRI (57.8%) occurred during the weekends, with a peak on Fridays (40.9%) followed by Saturdays (16.9%). A large proportion of injuries occurred between 12:01 and 18:00 h on Fridays (42.2%) and 00:01 and 6:00 h on Saturdays (50%) (Fig. 4c). From 2012, there was a definite trend toward an increase in the proportion of female ATVRIs (Fig. 2b). These trends were sustained in subsequent reports submitted to the Traffic Department of the Ministry of Interior for the years 2018–2021.

**Fig. 5:**
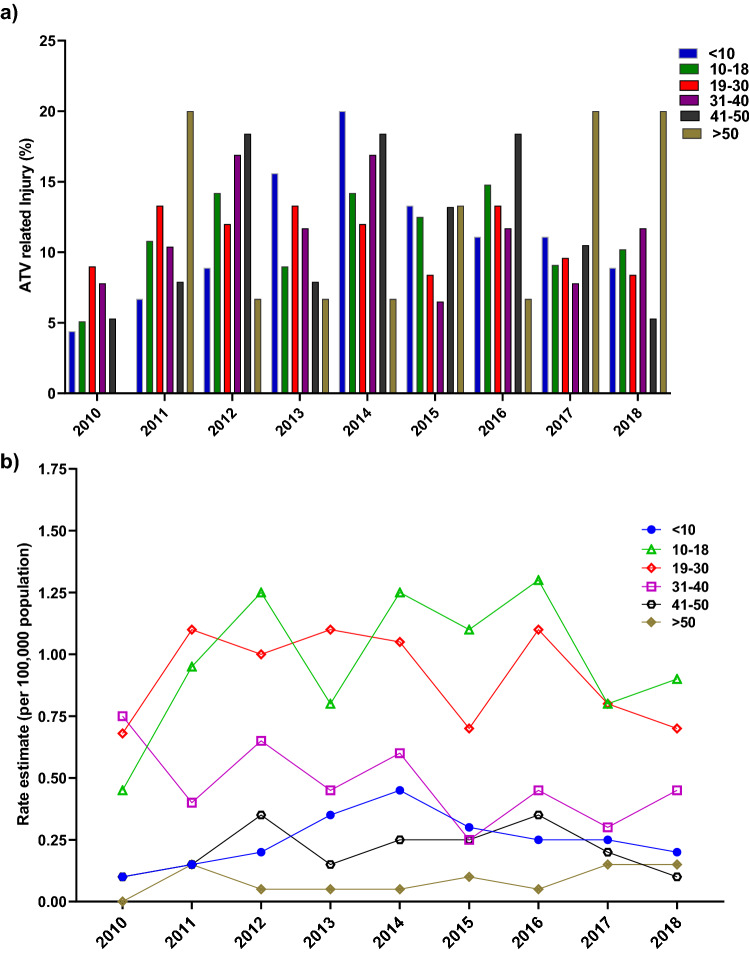
**a** ATV-related injuries by different age groups (2010–2018); **b** annual estimated injury rates per 100,000 persons within each age group

Figure 5a shows the breakdown of age groups’ estimated numbers of ATV-related emergency department-treated injuries from 2010 to 2018. The Pediatric age group exhibited an increasing trend toward ATV-related injuries between 2013 and 2016. The two oldest age groups (41–50 and > 50) had more significant injury estimates in 2017 and 2018, in contrast to all younger age groups (under age 10 and 10–18), which decreased between 2017 and 2018. Differences in population size among age groups and time likely influence the number of injuries for each age group. Figure 5b represents the comparisons normalized by population size and presents the annual estimated injury rates per 100,000 persons within each age group from 2010 to 2018.

### Predictors for head injury and injury severity among ATV users

Multivariate logistic regression analysis showed that young age (OR 0.95; 95% CI 0.93–0.98; *p* = 0.001) and ISS (OR 1.2; 95% CI 1.16–1.26; *p* = 0.001) were independent predictors of head injury after adjusting for gender (OR 0.96; 95% CI 0.51–1.79; *p* = 0.90) and use of helmet (OR 0.38; 95% CI 0.08–1.75; *p* = 0.21). Age was not a predictor of severe overall body injury (ISS ≥ 16) (crude OR 0.99; 95% CI 0.98–1.01; *p* = 0.73).

## Discussion

ATV-related injuries could result in significant morbidity, mortality, and healthcare costs worldwide [11, 22–24]. There is a lack of up-to date representative research on the trends and patterns of ATV-related injuries in the Middle East. The study presents a large descriptive cohort of a 9-year representative sample of ATVRIs (*n* = 521) from the national trauma center in Qatar; that treats an average of 58 patients with moderate to severe ATVRIs every year. We identified the high-risk groups and periods with the highest risk for severe ATVRIs and recommended targeted and proven interventions to reduce this risk. The study showed that young age and ISS were independent predictors of head injury among ATV users. However, age was not a predictor of the severe overall body injury.

The most exciting findings are related to the age involved in the ATVRIs. However, there is inconsistency in the literature that defines the age group limits. Children (≤ 18 years) make up 15% of the Qatar general population; however, 40% of ATVRIs in the present cohort are children. Previous studies have reported that children under the age of 16 are responsible for 30 to 50% of all the ATVRIs and 35% of all ATV-related deaths [23, 25]. A similar study from Canada reported that children in the 10–14 age group contributed 45% of the ATVRIs [26]. Adil et al. [15] reviewed a single-center experience in the UK with ATVRIs over 6 years (*n* = 65 patients); in which 52% of ATV victims were children (< 18 years).

Currently, local regulations for ATV driving are lacking in our community. In many countries like the UK, it is illegal to carry a child on ATV, and children < 13 years are not allowed to use off-road ATV, and for on-road riding, a valid driving license is required [27]. Data from Canada and Australia recommend that children below 16 years not ride quad bikes [18, 26]. The Orthopaedic Trauma Association and the Pediatric Orthopaedic Society of North America recommend that children under the age of 12 should not use ATV [28]. Prior data from the USA showed that 22% of the ATV-related deaths occurred in children under the age of 16 [28].

The use of protective devices, particularly helmets, is well known to reduce head injuries following ATV crashes [29]. Proper helmet use is estimated to reduce head injury risk by 64% and death by 42% [10, 29, 30]. Furthermore, it has been demonstrated that wearing a helmet while riding an ATV reduces intracranial hemorrhage from 22 to 3%.[30]. Our database documented that 4% of patients with ATVRIs were wearing a helmet at the time of injury. Nonetheless, compliance with wearing a helmet is low in the published literature [19, 23, 27, 31, 32]. Poor documentation or lack of reinforcing laws can partially explain the observed low compliance for helmet use in our study and the published data [19, 23]. Moreover, Merrigan et al. reported a trend of underutilizing the helmet for passengers compared to drivers (19% vs 35% respectively) [33].

Females make up only 25% of the general population of Qatar, however the  latest report to the Traffic Department demonstrated that almost 40% of all victims were females. Regarding gender, the majority of ATVRIs were males, though; we observed an increase in the percentage of girls compared to earlier years of this observational study. Interestingly, girls have begun participating in more outdoor recreational activities. In the current study, they were equally averse to wearing protective gear as their male counterparts, making them vulnerable to injuries and fatalities.

The apparent seasonal and weekends trend toward a higher incidence of ATVRIs reflect the increase of the ATV use for recreation and leisure. More than 57.8% of all ATVRIs occurred on weekends, with a peak on Fridays (40.9%; 12:01–18:00 h) followed by Saturdays (16.9%; 00:01–6:00 h).

One in 50 will die from ATVRIs, and at least 1 in 13 will be left with a lifelong disability. The overall fatality rate for ATV was 1.1 per 100,000 population. Upperman et al. found an overall fatality rate of 0.09 ATV deaths per 100,000 populations among children under 16 from 1982 to 1998 [34]. Another study, conducted by Helmkamp and colleagues (2000–2005), found 1043 ATV deaths in children under the age of 16 (a rate of 0.27 per 100,000 populations), compared to 4161 deaths in adults (a rate of 0.31 per 100,000 population) [32]. Compared to previous studies, our study shows a significant increase in ATV-related fatality rates between 2010 and 2018. During this period, the popularity of ATVs has skyrocketed [6, 7, 9, 16]. While much attention has been on pediatric trauma associated with ATV use, these vehicles are equally unsafe for riders regardless of their age.

This study has many limitations, including the retrospective design and the lack of documentation regarding ATV-specific information (involving the type, number of wheels, engine size, and crush protection devices). The age of 4 victims remained unknown (three of them died on arrival). The location of these crashes (street, personal residence, public area, farm, or private land), as well as the exact mechanism involved (rollover, collision (ATV vs ATV or Vehicle), fall from ATV, driver or passenger or trapping underneath), are not documented in the database. We only analyzed the contribution of admitted patients to the extent of the ATV problem, those who sustained minor injuries or injuries that did not necessitate admission were not captured. Also, we lacked information about the victim’s status as a tourist, and we believe that the majority were residents of Qatar. Finally, prehospital deceased were not analyzed. However, we highlight the significant injuries related to ATVs’ usage- over 9 years which is the largest cohort reported in the Middle East.

We have recently started a prospective ATV registry with concurrent patient interviews to explore the problem further and document more details on the mechanism, beliefs, and behaviors. These vehicles need advanced psychomotor skills (coordination, muscle strength, and judgment) and are unsuitable for operation or use by children and inexperienced adults. The native population is disproportionately affected by ATV injuries and should be the focus on awareness, enforcement, strict governance, and safety campaigns. Compliance with helmet use and safe riding behavior is low and calls for legislation and public awareness strategies. Information campaigns must target families, parents and children with age and culturally appropriate media and include the ATV rental and sales outlets. The focus is delivering key messages about helmet use and age restriction right at the start of the school year to address high-risk populations in September before the winter camping season to address the high-risk season of ATVRIs.

We strongly recommend that national government officials [or policy makers] recognize that ATV injuries have at least four times the death and disability rates in Qatar when compared to other high-income countries. ATVRIs disproportionately affect children and the local population and that this purely recreational activity should not be allowed to proceed in a largely unregulated manner. The consistent enforcement of existent rules that restrict usage of ATVs by user age and safe zones and the creation of new legislation that will require the use of helmets and other personal protective equipment should be prioritized.

Overall, we believe that this study should serve as a reference for public policymakers regarding safe ATV use in the state of Qatar and that it should contribute to the growing body of evidence that informs the creation of new ATV legislation.

## Conclusions

This is a nationwide study looking at all age groups sustaining ATV-related trauma in Qatar. ATV-related injuries were observed in all age groups following leisure and recreation use. It follows a seasonal pattern, and there is an immediate need to reinforce helmet use and raise public awareness. These data can be used as a guide and reference for better prevention strategies and future research.

## Supplementary Information

Below is the link to the electronic supplementary material.

Supplementary file1 (DOCX 39 kb)

## Data Availability

All relevant data were given in the result, tables, and figures in this manuscript.
