# Correction to: All-terrain vehicle (ATV)-related injuries among different age groups: insights from a 9-year observational study

**DOI:** 10.1007/s00068-022-02028-4

**Published:** 2022-07-05

**Authors:** Husham Abdelrahman, Naushad Ahmad Khan, Ayman El-Menyar, Rafael Consunji, Mohammad Asim, Mushrek Alani, Adam Shunni, Abubaker Al-Aieb, Hassan Al-Thani

**Affiliations:** 1 Trauma Surgery Section, Hamad Medical Corporation & Weill Cornell Medical College, Hamad General Hospital (HGH), PO Box 3050, Doha, Qatar; 2 Clinical Research, Trauma and Vascular Surgery Section, HGH, Doha, Qatar; 3 Clinical Medicine, Weill Cornell Medical College, Doha, Qatar; 4 Trauma Surgery Section, Hamad Injury Prevention Program, HGH, Doha, Qatar

## Correction to: European Journal of Trauma and Emergency Surgery 10.1007/s00068-022-01984-1

In this article Fig. 2 is incomplete in the PDF. The Fig. 2 should appear as shown below.

**Fig. 2:**
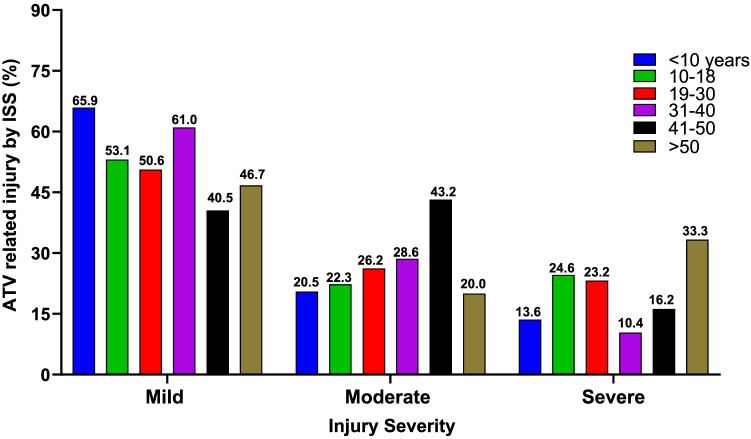
The proportion of ATV-related injuries stratified by injury severity defined by injury severity score (ISS)

The original article has been corrected.

